# Beatquency domain and machine learning improve prediction of cardiovascular death after acute coronary syndrome

**DOI:** 10.1038/srep34540

**Published:** 2016-10-06

**Authors:** Yun Liu, Benjamin M. Scirica, Collin M. Stultz, John V. Guttag

**Affiliations:** 1 Institute for Medical Engineering and Sciences, Massachusetts Institute of Technology (MIT) and Harvard-MIT Division of Health Sciences and Technology, 77 Massachusetts Ave. Cambridge MA 02139, USA; 2 TIMI Study Group, Cardiovascular Division, Department of Medicine, Brigham and Women’s Hospital, and Harvard Medical School, 75 Francis St., Boston Ma. 02115, USA; 3 Department of Electrical Engineering and Computer Science, MIT, 77 Massachusetts Ave. Cambridge MA 02139, USA; 4 Computer Science and Artificial Intelligence Laboratory, MIT, 77 Massachusetts Ave. Cambridge MA 02139, USA.

## Abstract

Frequency domain measures of heart rate variability (HRV) are associated with adverse events after a myocardial infarction. However, patterns in the traditional frequency domain (measured in Hz, or cycles per second) may capture different cardiac phenomena at different heart rates. An alternative is to consider frequency with respect to heartbeats, or beatquency. We compared the use of frequency and beatquency domains to predict patient risk after an acute coronary syndrome. We then determined whether machine learning could further improve the predictive performance. We first evaluated the use of pre-defined frequency and beatquency bands in a clinical trial dataset (N = 2302) for the HRV risk measure LF/HF (the ratio of low frequency to high frequency power). Relative to frequency, beatquency improved the ability of LF/HF to predict cardiovascular death within one year (Area Under the Curve, or AUC, of 0.730 vs. 0.704, p < 0.001). Next, we used machine learning to learn frequency and beatquency bands with optimal predictive power, which further improved the AUC for beatquency to 0.753 (p < 0.001), but not for frequency. Results in additional validation datasets (N = 2255 and N = 765) were similar. Our results suggest that beatquency and machine learning provide valuable tools in physiological studies of HRV.

Frequency domain measures of heart rate variability (HRV)^1^ identify patients who are at increased risk of adverse medical outcomes^2^,^3^,^4^,^5^,^6^,^7^. Most frequency domain measures are in units of cycles per second (Hz). However, “frequency” can also be measured with respect to heartbeats, in units of cycles/beat – which is sometimes expressed as “equivalent Hz” (cycles/beat * mean heart rate) in the literature^8^,^9^,^10^,^11^,^12^,^13^,^14^,^15^,^16^. In this work, our goal is to compare these two frequency domains in the context of predicting cardiovascular death after a non-ST-elevation acute coronary syndrome (NSTEACS).

To illustrate the difference between frequency (Hz) and beatquency (cycles/beat), assume that there are two patients with constant heart rates of 60 and 120 beats per minute respectively. A frequency of 0.5 Hz corresponds to every 2 beats in the first patient but every 4 beats in the second. Use of the beatquency domain may result in more consistent predictive frequency bands between patients with different heart rates, and across different times for a given patient.

As recommended by the HRV Task Force^1^, most studies that analyze frequency domain HRV do so in the frequency domain. Interestingly, however, the beatquency domain is associated with greater intra-subject and inter-subject consistency in humans^8^ as well as across rats and dogs^15^. Remarkably, to our knowledge, there have been no published studies of the utility of beatquency in predicting adverse outcomes.

In this study we tested the hypothesis that beatquency applied to an established HRV measure, Low Frequency High Frequency (LF/HF, a ratio of energy in two bands)^1^ will improve prediction performance for cardiovascular death post NSTEACS. We focused on frequency domain measures and LF/HF because LF/HF was the best performing HRV measure on our datasets relative to other frequency domain measures (e.g., the Standard Deviation of normal RR intervals, or SDNN) in our datasets^17^,^18^. Because these original frequency bands were not explicitly selected for optimal risk stratification, we further used machine learning to discover the frequency and beatquency bands most useful in predicting cardiovascular death post NSTEACS.

## Clinical Data and Outcomes

Our work primarily utilized electrocardiographic (ECG) recordings obtained from a clinical trial of patients after NSTEACS^19^. The dataset consists of all 2,302 patients in the placebo arm, and contains 93 cardiovascular deaths within the median follow-up of one year. We focused on the placebo arm because the treatment arm was prescribed ranolazine, a drug that may have anti-arrhythmic properties^20^ and thus affect ECG measures. We used this dataset to compare frequency and beatquency LF/HF and to train and test machine learning models. If not otherwise indicated, all results in this work refer to this dataset. In addition, we employed two additional “holdout datasets”, for further validation of the machine learning models that were developed, using the dataset described above. These datasets are described in the Supplementary Information. Patient characteristics are reported in Table 1. For all three datasets, up to 7 days of ambulatory ECG signals recorded at 128 Hz are available for each patient. In this work, we used the first 24 hours from each patient to compute the heart rate time series. The protocol was approved by the local or central Institutional Review Board at all participating centers.

## Results

### LF/HF in Frequency and Beatquency

**Figure 1:**
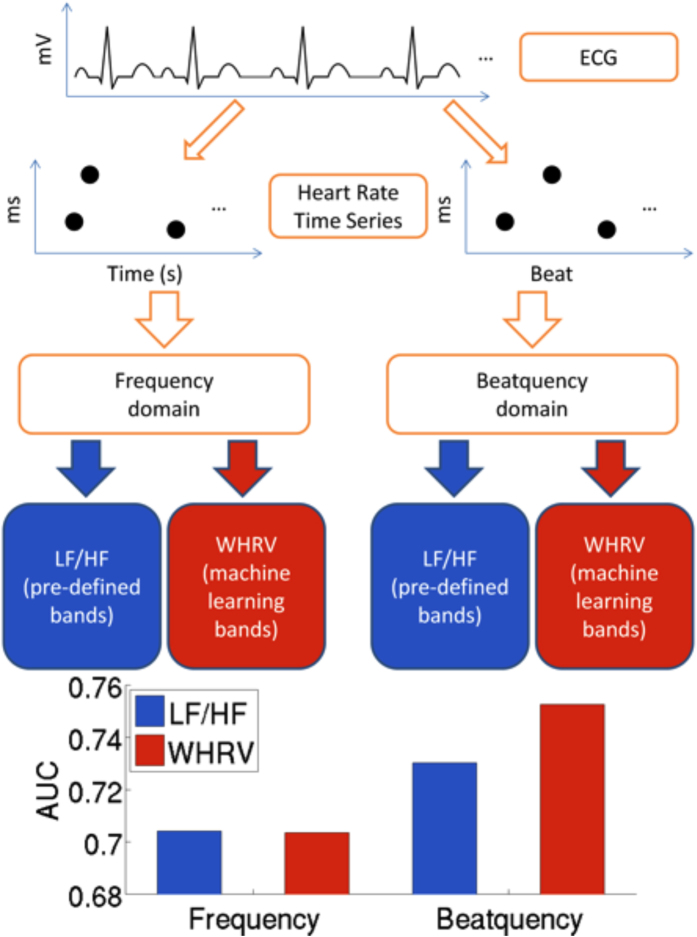
Schematic of calculation of heart rate variability (HRV) metrics in frequency and beatquency. The input ECG signal is used to compute the heart rate time series with time and beat index as the x-axis. Next, this heart rate time series is converted to the frequency or beatquency domain. The final metrics measure the energy in pre-defined bands (Low Frequency High Frequency, LF/HF), or weighted by machine learning (Weighted HRV, WHRV).

The frequency domain quantifies changes that are periodic with respect to time, while the beatquency domain quantifies changes that are periodic with respect to heartbeats. For example, a beatquency of 0.5 cycles/beat quantifies events that alternate with each heartbeat. In terms of frequency, this would correspond to 0.5 Hz at a heart rate of 60 beats per minute, but 1 Hz at a heart rate of 120 beats per minute. The calculation of frequency and beatquency domain metrics is schematically outlined in Fig. 1. The initial steps of both calculations are similar: we obtained a heart rate time series, divided it into 5-minute windows, and converted each window into each of the two frequency domains. For the “pre-defined bands” calculation (blue in lower half of Fig. 1), we computed the *frequency LF/HF* using the established LF (0.04 to 0.15 Hz) and HF (0.15 to 0.40 Hz) bands^1^. Diagnostic bands for *beatquency LF/HF* were based on a prior study that applied LF and HF beatquency bands to normal adults as well as those with coronary artery disease and congestive heart failure: 0.03 to 0.14 (LF band) and 0.14 to 0.40 cycles/beat (HF band)^14^.

The Area Under the Curve (AUC) for LF/HF in frequency and beatquency in the entire cohort were significantly different, 0.713 for frequency and 0.731 for beatquency, p < 0.01 (the receiver operating characteristic curves are shown in Supplementary Fig. S1). Using the quartile as high-risk cutoffs for time and beat frequency (0.841 and 0.832 respectively) resulted in hazard ratios (HRs)^21^ of 3.7 and 4.6. After adjusting for the Thrombolysis in Myocardial Infarction Risk Score (TRS)^22^, the HRs were 3.0 and 3.9 respectively. After adjusting for TRS, ejection fraction (EF), and B-type natriuretic peptide (BNP)^23^, both HRs were 2.4. All HRs were statistically significant (p < 0.01). The category-free net reclassification improvement^24^ for beatquency relative to time frequency was 75% (95% confidence interval 42–93%).

### Machine Learning (WHRV) in Frequency and Beatquency

The LF and HF frequency and beatquency bands above were not optimized for prediction of adverse events, but were instead based on previous physiological studies and observations of where peaks are located in the power spectra. In this section, we evaluate a machine learning procedure to learn the predictive frequency and beatquency bands as well as the relative importance (weights) of each band. We term this approach Weighted HRV (WHRV), and outline the approach in red in the lower half of Fig. 1.

In the WHRV approach we first obtained a heart rate time series, divided it into 5-minute windows, and converted each window into each of the two frequency domains. Unlike the computation of frequency LF/HF where we computed the energy in pre-specified bands, here we estimated the power spectral density from 0.01 to 0.50 Hz in 0.01 increments – yielding 50 frequency bands. An identical approach applied to the heart rate time series with “beats” on the x-axis yields 50 beatquency bands ranging from 0.01 to 0.50 cycles/beat. In each case, these 50 values formed a feature vector for each patient. We first applied unsupervised approaches (Methods) to understand if dimensionality reduction based on these frequency and beatquency features would reveal differences between the two types of frequency (Table 2). Since we tested several different unsupervised methods for assessing patient risk (e.g., centered and non-centered PCA and MCE), we only report the AUCs for the method that yielded the best results in Table 2. (Details of the performance of each of the methods we investigated are shown in Supplementary Figs S3–S5). In all cases (particularly in the larger validation dataset D2), the discriminatory performance was improved in beatquency relative to frequency (see Table 2). Furthermore, these data demonstrate that these techniques can perform remarkably well, yielding AUCs as high as 0.726 in the larger validation dataset D2 (using principle component analysis), and as high as 0.767 in the smaller dataset D3 (using minimum curvilinear embedding^25^). The fact that dimensionality reduction using beatquency features can yield improved performance argues that a combined approach that uses both a dimensionality reduction approach coupled with supervised learning may further improve the performance of the beatquency approach.

Based on these encouraging findings, we adopted a supervised learning approach by feeding the feature vectors to an L1-regularized logistic regression algorithm, which selects the most important features (frequency or beatquency bands). The algorithm outputs a weight vector, which quantifies the relative importance of each frequency band in the prediction. For each patient, the WHRV is the scalar valued dot product of the feature vector with the weight vector, and higher values indicate higher risk. The machine learning protocol is described in more detail in the Methods.

We trained separate models on each of 1,000 randomly chosen training sets and evaluated the results averaged across the 1,000 test sets to ensure results were not dependent on the randomness of each split (Methods). The AUC for beatquency WHRV was significantly higher than that of frequency in the test sets taken from dataset D1 (Table S2, 0.753 versus 0.704, p < 0.001). Applying the same trained models to two additional holdout sets demonstrated similar trends (0.728 versus 0.691, and 0.738 versus 0.679, p < 0.001, see Table 2). Moreover, while machine learning left the AUC of frequency LF/HF unchanged, it increased the AUC of beatquency LF/HF using test sets from dataset D1 (0.704 to 0.730, p < 0.001, Table S2) and in the validation sets (Table 2). These values are significantly higher than those of another HRV metric, SDNN (standard deviation of NN intervals).

Lastly we note that since the AUC may be deceptive in the setting of significant class imbalance (i.e., a relatively small number of positive examples)^16^,^25^,^26^,^27^, we also calculated the AUPR for each of the predictive methods we examined (Table 3). While the AUPRs on the smaller validation dataset, D3, do not show an improvement in performance when a beat-space analysis is used, in most cases the performance on the larger dataset, D2, shows an improvement in the beatquency domain. The fact that the overall AUPRs are low highlights the fact that there is room for improvement with respect to development of a model with true clinical utility. Nevertheless, we were encouraged by the fact that beatquency improves the AUC in all of our applied methods.

To further explore the utility of our methods, we evaluated them on patients who would not be identified as high risk by TRS (TRS ≤ 4). The AUC for beatquency WHRV increased to 0.773 in this population, and this performance was preserved in both males (0.772) and females (0.758) (Table S2). Similarly, these AUCs were higher than that those in frequency, and substantially higher than SDNN.

We present the HRs of the models before and after adjusting for clinical variables in Table 4. The unadjusted HR’s were 3.70 and 5.19 for frequency and beatquency respectively. In multivariable analysis, the HRs were 3.13 and 4.38 after adjusting for TRS, and 1.99 and 2.91 after adjusting for TRS, EF and BNP. After adjusting for all three variables, the WHRV beatquency metric had a confidence interval that excluded 1. In addition, the adjusted HR using machine learning was higher than those using pre-defined bands for beatquency; i.e., the LF/HF beatquency HR is 2.45 vs. a beatquency WHRV HR of 2.91 (where only the latter has a 95% CI that excludes 1). This was not the case when machine learning was applied to the frequency domain; i.e., the LF/HF frequency HR is 2.28 vs. a WHRV frequency HR of 1.99.

The results above used the quartile as the high-risk cutoff. To explore the utility of using other cutoffs, we compared the positive predictive values and negative predictive values of these four metrics over all possible cutoffs from the 5^th^ to the 95^th^ percentile, in increments of 10 percentiles (Supplementary information, Figs S7 and S8). Beatquency WHRV demonstrated the highest positive predictive value from the 25^th^ to the 85^th^ percentile, demonstrating robustness to the choice of the cutoff. The negative predictive values of all the measures were above 96% at all cutoffs, suggesting utility in identifying patients who are not at high-risk.

**Figure 2:**
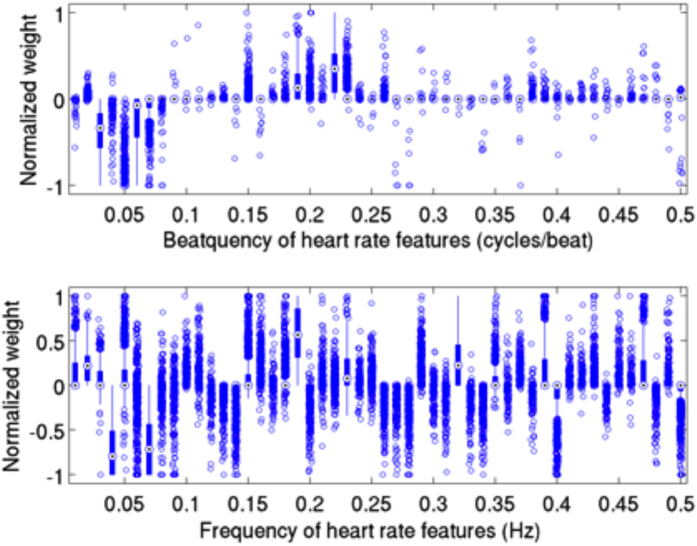
Boxplot of normalized weights of frequency and beatquency machine learning models trained on the same 1,000 training splits. Quartiles are represented by the edges of lines, box, and central dot, while circles indicate outliers. The average standard deviations of the weights are 0.057 in beatquency and 0.153 in frequency.

Each of the 1,000 randomly chosen training sets generated a distinct weight vector. We observed a greater consistency in beatquency features compared to frequency in these 1,000 weight vectors: the average standard deviation of the normalized beatquency feature weights is less than half that of the frequency value (Fig. 2, 0.057 versus 0.153, p < 0.001). Moreover, beatquencies between 0.03–0.07 cycles/beat have consistently negative weights across the 1,000 different models – suggesting that high power in these bands is associated with decreased rate of one-year cardiovascular death. By contrast, beatquencies between 0.18–0.24 cycles/beat are associated with positive weights – suggesting that high power in these bands is associated with an increased rate of one-year cardiovascular death. Since the models built using frequency features show much more variation across the 1,000 different models, it is difficult to make reliable inferences about the relative importance of different frequency bands.

## Discussion

Since the autonomic nervous system is an important regulator of heart rate, analysis of heart rate variability can provide useful information about autonomic tone. Quantitative metrics like HRV that provide insight into the autonomic tone are often calculated using the frequency domain. Frequency domain HRV metrics have been shown to provide useful prognostic information after a myocardial infarction in multiple studies^2^,^3^,^4^,^5^,^6^,^7^ and after NSTEACS^17^,^18^. However, though frequency HRV measures are associated with elevated risk after NSTEACS, these risk measures miss significant numbers of deaths^18^. Our results suggest that quantifying LF/HF in terms of beatquency instead of frequency improves the ability to identify high-risk patients after a NSTEACS.

**Figure 3:**
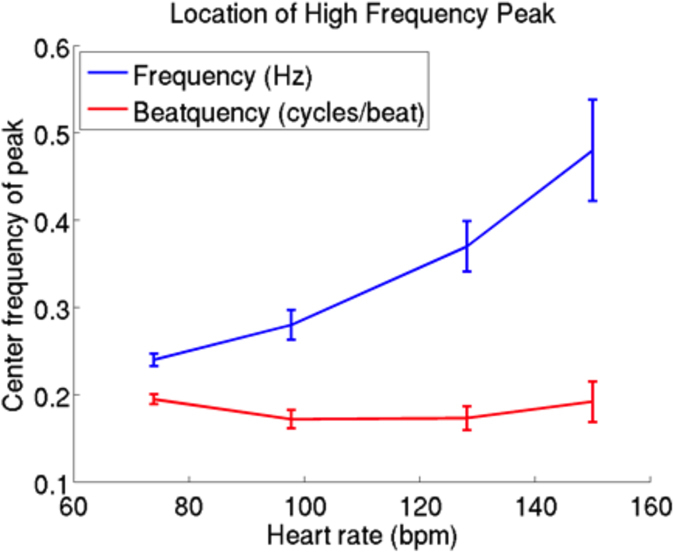
Location of high frequency band in exercising subjects. Frequency data are from ref. 28; beatquencies are estimated by dividing the frequency value by the heart rate in Hz. Error bars represent standard error.

Analysis of the relationship between the frequency spectrum and the average heart rate provides insight into the improved performance of measures that are calculated from the beatquency spectrum. In a study by Perini *et al*., young male subjects exercised on a cycle ergometer at pre-specified exercise intensities^28^. With increasing exercise intensity, the center frequency of the HF band increased from 0.24 to 0.48 Hz (Fig. 3). By contrast, our estimate of the center frequency of the corresponding HF band in beatquency varies from 0.17 to 0.19 cycles/beat over the same range of heart rates (Fig. 3). Because beatquency bands are less sensitive to changes in average heart rate, they allow patients and ECG segments with different average heart rates to be directly compared.

Because the location of the HF band is influenced by the respiratory rate^29^,^30^, and heart rate and respiratory rate are correlated, beatquency may be correcting for respiratory rate. There exist sophisticated algorithms to extract the respiratory rate from the ECG, for example in ref. 31, and to adjust the frequency bands based on the respiratory rate^32^,^33^. These methods may improve risk stratification further, however we note that the simplicity of beatquency enables it to be easily applied to existing large HRV datasets and also to new HRV studies without additional equipment or expert oversight in selecting appropriate frequency bands.

The fact that beatquency WHRV (using machine learning) outperformed beatquency LF/HF (using pre-defined bands) highlights the benefit of adopting a data-driven, machine learning approach to analyzing heart rate spectra. An analysis of the weights of the beatquency bands finds trends that are similar to what has been observed with trends in standard frequency LF/HF measurements. In particular, the signs of the beatquency bands weights (negative at 0.03 to 0.07 cycles/beat and positive at 0.18 to 0.24 cycles/beat) are consistent with the notion that in patients post myocardial infarction, lower values of LF/HF are associated with adverse events in some patient populations^34^. Our data also demonstrate that using features derived from the beatquency spectrum yields logistic regression models that have improved performance relative to models derived from the frequency spectrum. Thus beatquency may also have utility in other machine learning models that leverage frequency domain ECG features, such as for arrhythmia classification^35^. One limitation of our observations is that we used Holter data that were collected at the standard frequency of 128 Hz. Higher sampling rates may improve the accuracy of our measured RR intervals and further improve results, especially for standard frequency domain HRV measures.

Applying machine learning to frequency bands does not yield a significant improvement in either the AUC or the unadjusted or adjusted hazard ratios relative to the pre-defined frequency bands. That machine learning improves risk stratification with beatquency bands but not frequency bands can be explained by the greater consistency of feature weights in beat- compared to frequency. Since differences in average heart rate are associated with shifts in the frequency domain spectra, the predictive frequencies may differ between patients and across time for each patient. Thus frequency features may not *generalize* across patients or even between segments of different heart rates for the same patient. This leads to unchanged or even decreased performance despite an attempt to learn the predictive frequencies.

Overall, applying beatquency and machine learning to heart rate variability metrics improves our ability to identify patients at elevated risk of cardiovascular death post NSTEACS. Our machine learning approach in beatquency also reveals predictive bands that can be studied in more detail in physiological studies, and more generally, showcases a data driven approach to selecting frequency bands for analysis. Our discriminatory results could be improved further by including other variables (both based on HRV and otherwise) in the machine-learning model. As already mentioned, the objective of this work was to compare the ability of two different methods for representing the heart rate time series. Our data demonstrate the relative power of a beatquency approach for identifying patients at high risk of death post ACS. Effective risk metrics may arise from models that combine beatquency variables with other non-linear metrics that have been applied medical data^36^,^37^,^38^. Beyond predicting patient risk, future work would be required to further validate these risk metrics and to find the most effective therapies in high risk patients highlighted by these risk metrics.

## Methods

### Frequency LF/HF

We first segmented the 24-hour long ECG signal to find individual heartbeats, and used the Signal Quality Index^39^ to extract the normal beats. To ensure that only normal beats were analyzed, the two beats adjacent to each abnormal beat were removed as well. We performed manual checks to ensure these segmentation and beat classification steps were appropriate. These steps are described in detail in our previously published work^40^.

To calculate the frequency LF/HF we divided the heart rate time series into 5-minute intervals and estimated the power spectral density in the frequency domain. There are many ways to compute the frequency domain power spectrum; we use the Lomb-Scargle periodogram, which provides a natural way to handle unevenly sampled data^41^,^42^. For the RR time series, this uneven sampling has two sources: the inherent heart rate variations in the underlying ECG, and the removal of ectopic beats or artifacts. We then computed the ratio of the energy in the LF band to the HF band for each 5-minute window, and defined the median value of all these LF/HF ratios to be the LF/HF for that patient. A low LF/HF ratio is known to be associated with poorer prognosis^34^.

### Beatquency LF/HF

*Frequency LF/HF* was obtained from the power spectrum of the heart rate time series, where the time points of the beats were used as a temporal reference in the Lomb-Scargle periodogram. *Beatquency LF/HF* was also obtained from the power spectrum of the heart rate time series, however, the heartbeat indices were used as the “temporal” reference. Where beats or parts of the signal were removed (as described in the previous section), the number of removed beats was estimated using the average time intervals of the heartbeats immediately adjacent to the gap^18^. As mentioned in the results section, diagnostic bands for beatquency LF/HF were based on a prior study that applied LF and HF beatquency bands to normal adults as well as those with coronary artery disease and congestive heart failure: 0.03 to 0.14 (LF band) and 0.14 to 0.40 cycles/beat (HF band)^14^. The remaining steps in beatquency LF/HF computation were identical to the frequency construction. Figure 1 compares the two approaches.

We verified that frequency and beatquency bands were meaningfully different by computing the correlation coefficient of the LF and HF bands in frequency with bands in beatquency. The most similar beatquency bands had correlations of 0.63 (0.04 to 0.13) and 0.81 (0.10 to 0.37) for frequency LF and HF respectively. These correspond to 40% and 66% of the explained variance, respectively.

### Machine Learning

In this section, we describe a machine learning procedure to learn predictive frequency and beatquency bands as well as the relative importance (weights) associated with each band. First, we will describe the feature construction for each patient, where each feature is related to a frequency or beatquency band. These features were then used to train a model, which outputs a weight vector and a prediction for each patient. The weight vector quantifies the importance of each frequency band in the prediction, and for each patient the prediction was the dot product of the feature vector with the weight vector. We term the resulting scalar-valued prediction the Weighted HRV (WHRV).

The initial steps were similar to the LF/HF computation (Fig. 1). We obtained a heart rate time series, divided it into 5-minute windows, and converted each window into the frequency domain. Unlike the computation of frequency LF/HF where we computed the energy in pre-specified bands, here we estimated the power spectral density from 0.01 to 0.50 Hz in 0.01 increments. After this was done for all 5-minute windows for that patient, we defined each feature as the 90^th^ percentile of the power for that frequency over all 5-minute windows. This 90^th^ percentile value was used in previous ECG measures, and is intended to capture the maximum variability in a particular frequency while excluding outliers^17^,^18^. These 50 features quantify the heart rate variability at each frequency from 0.01 to 0.50 Hz.

The beatquency features were constructed in a similar fashion, except that beat indices were used as the temporal reference in the Lomb-Scargle periodogram instead of time points. The other steps of preprocessing (i.e., division into 5-minute windows, and taking the 90^th^ percentile) were identical. The 50 features obtained quantify the heart rate variability in 50 beatquency bands from 0.01 to 0.50 cycles/beat. We visualized these frequency and beatquency features using principle component analysis (PCA) and non-centered Minimum Curvilinear Embedding (MCE, a type of topological-based nonlinear kernel PCA^25^, and found promising discriminatory performance (Tables 2 and 3, Supplementary Figs S3–S5). These unsupervised results encouraged us to apply supervised machine learning methods, as described in the next sections.

The machine learning protocol consists of randomly dividing the initial dataset into a training set and a test set in a 2:1 ratio, stratified by outcome; i.e., training sets and test sets have the same percentage of cardiovascular deaths. A L_1_-regularized logistic regression model (Equation 1) as implemented in LIBLINEAR^43^, was then trained on the training set and tested on the test set.


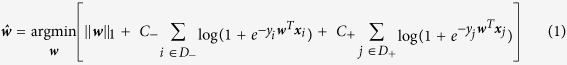


where 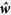 is the optimum weight vector that minimizes the expression in square braces in equation 1, ||•||_1_ is the L_1_ norm, D_−_ and D_+_ are data (patients) in the negative (alive) and positive (dead) classes, C_−_ and C_+_ are cost parameters for the two classes, y_*i*_ and y_*j*_ are the scalar class labels for patients *i* and *j*, and assumes a value of either −1 or +1. The variables **x**_*i*_ and **x**_*j*_ denote the feature vectors for patients *i* and *j*, respectively.

Because we expected only a relatively small subset of the frequency domain to contain pertinent prognostic information, we applied L_1_ regularization to perform implicit feature selection. Within the training set, five repeats of 5-fold cross validation were used to optimize the cost parameters C_−_ and C_+_, for patients in the negative (alive) and positive (dead) classes respectively. This asymmetric cost was necessary because only 4% of the patients experienced an event. C_−_ is optimized from the range 10^−6^ to 10^−2^ in exponential steps, while keeping C_+_ = k * C_−_, where k is the class imbalance ratio. We standardized each frequency domain feature by subtracting the mean value of that feature and dividing by its standard deviation across all patients.

### Evaluation of Machine Learning Models

We evaluated the performance of WHRV by randomly splitting the dataset into a training set and a test set in a 2:1 ratio. This training/test split procedure ensures that models are evaluated based on their performance on patients that the models have previously not “seen”. We trained a machine learning model on the training set and measured the discriminatory performance on the test set using the area under the receiver operating characteristic curve (AUC)^44^ for the outcome of cardiovascular death within 1 year. The AUC can be interpreted as a probability: given a patient who eventually experienced the outcome of interest (e.g. cardiovascular death within 1 year) and another who did not, what is the probability that the model ranks the patient who died as at a higher risk than the patient who did not^45^?

We repeated this training/test split 1,000 times to reduce the effects of selecting an overly optimistic or pessimistic test set, i.e. we constructed 1,000 training sets and 1,000 test sets. We report results averaged over the 1,000 test sets. To evaluate the performance of these models in the additional holdout datasets (as described in the supplement), we computed the AUC for each of the 1,000 models in the holdout dataset, and report the average AUC. In addition, we also evaluated the trained models on two additional datasets (reported in the Supplement) and summarize the results in the main text.

The relative importance of the 50 frequency and beatquency features were analyzed across the 1,000 different models by dividing the weight of each feature by the maximum absolute weight in each model (i.e., the largest absolute value of the normalized weights will be 1). We estimated the variation in feature weights by first computing the standard deviation of each normalized feature weight across all 1,000 models to obtain a standard deviation for each feature. We summarized these 50 standard deviations by taking the average. A higher value indicates a higher variation of feature weights across different training sets.

### Comparison with Clinical Measures

We evaluated the performance of our model relative to several clinical variables, the Thrombolysis In Myocardial Infarction Risk Score^22^ (TRS), left ventricular ejection fraction (EF), and B-type natriuretic peptide^23^ (BNP). The TRS summarizes the effects of numerous risk factors, including age, elevated biomarkers, and presence of coronary artery disease. The score is defined as the number of risk factors (out of 7) that a person has, and thus ranges from 0 to 7. Patients with TRS ≥ 5 are considered to be at high risk^22^. EF quantifies the fraction of blood ejected in each cardiac cycle; we use ≤40% as the threshold for high risk^18^. BNP is a blood marker indicating ventricular stretch, and >80 pg/ml is considered a high-risk cutoff 23. Because EF and BNP were only measured in 47% of the patients^18^, we build separate multivariable models: a model that includes only TRS and uses all the patients, and a model that includes TRS, EF, and BNP, and uses only the patients with measured values of both EF and BNP.

### Statistical Analysis

In the LF/HF section, we assess the statistical significance of differences between the AUCs of two metrics (frequency and beatquency versions) on the entire dataset^46^. In addition, we computed the category-free net reclassification improvement to assess the difference between the frequency and beatquency LF/HF^24^. In the machine learning section, we assess the difference of AUCs across 1,000 test sets using a two-tailed paired t-test. Because of the known issues with the AUC in imbalanced datasets^16^,^25^,^26^,^27^, we also report the area under the precision recall curve (AUPR). Because dataset D3 has *few outcomes* (distinct from *low proportion* of outcomes), flipping the prediction in a single positive patient can cause a large change in precision and recall and “raggedness” in the precision recall curve. Thus, we place more emphasis on performance measurements on the larger datasets D1 and D2.

In addition, we computed the hazard ratio (HR) of the different HRV risk metrics using the Cox proportional hazards regression model^21^. We dichotomized the continuous machine learning predictions at the upper quartile of each test set; thus the HR indicates the hazard ratio of the upper quartile compared to the remaining patients. We adjusted the HR for TRS, EF, and BNP using binary variables defined by the high-risk cutoffs described in the previous section. Thus, we present three HRs: unadjusted, adjusted for TRS, and adjusted for all of TRS, EF, and BNP.

## Additional Information

**How to cite this article**: Liu, Y. *et al*. Beatquency domain and machine learning improve prediction of cardiovascular death after acute coronary syndrome. *Sci. Rep.* **6**, 34540; doi: 10.1038/srep34540 (2016).

## Supplementary Material

Supplementary Information

## Figures and Tables

**Table 1:** Patient characteristics.

Dataset	D1	D2	D3
N	2302	2255	765
1-year cardiovascular death (CVD)	93 (4.0%)	77 (3.4)	14 (1.8)
Median follow-up	1 year	1 year	90 days
Age, years, median (IQR)	64 (55–72)	63 (55–72)	62 (54–71)
Age ≥ 75 (%)	17	17	14
Female (%)	35	34	36
Diabetes mellitus (%)	34	33	24
Hypertension (%)	74	73	69
Current smoker (%)	25	27	56
Previous myocardial infarction (%)	33	33	25

IQR indicates interquartile range.

**Table 2:** Prediction performance (AUC, area under receiver operating characteristic curve) for cardiovascular death.

Dataset	D2		D3	
Type of frequency	Beat	Time	Beat	Time
LF/HF	**0.725**	0.710	**0.733**	0.709
PCA	**0.726**	0.620	**0.680**	0.669
MCE	**0.687**	0.652	**0.767**	0.713
WHRV*	**0.728**	0.691	**0.738**	0.679
Random	0.500			

Bold indicates the higher AUC in each pair (time vs beat).

^*^Indicates WHRV (the supervised approach) was trained on dataset D1 and the reported results are averaged over patients that the model was not trained on.

**Table 3:** Prediction performance (AUPR, area under precision recall curve) for cardiovascular death.

Dataset	D2		D3	
Type of frequency	Beat	Time	Beat	Time
LF/HF	**0.080**	0.076	0.054	**0.064**
PCA	**0.070**	0.058	0.072	**0.085**
MCE	0.067	**0.070**	0.066	**0.071**
WHRV*	**0.080**	0.067	0.050	**0.054**
Random	0.034		0.018	

Bold indicates the higher AUC in each pair (time vs beat).

^*^Indicates WHRV (the supervised approach) was trained on dataset D1 and the reported results are averaged over patients that the model was not trained on.

**Table 4:** Hazard Ratio (HR) averaged over 1,000 test sets in D1.

	WHRV HR (95% CI)		LF/HF HR (95% CI)	
	Beat	Time	Beat	Time
Unadjusted	**5.19 (2.60,9.45)**	3.70 (1.98,6.76)	4.44 (2.51,7.92)	3.73 (2.20,6.68)
Adj. for TRS	**4.38 (2.23,8.62)**	3.13 (1.72,5.89)	3.74 (2.01,7.07)	3.09 (1.73,5.84)
Adj. for TRS,EF,BNP†	**2.91 (1.05,8.68)**	1.99 (0.69,5.73)	2.45 (0.98,6.90)	2.28 (0.86,6.72)

95% confidence intervals (CI) reported in parenthesis. Bold indicates the highest HR in each row (p < 0.001 for unadjusted and adjusted for TRS; in the last row only WHRV in beatquency has a CI that does not include 1).

^†^In 7 out of 1,000 test sets, less than 30% of the patients had measured values of both EF and BNP, and therefore these test sets were excluded.
