# Supplementary material for: Beatquency domain and machine learning improve prediction of cardiovascular death after acute coronary syndrome
Source: Sci Rep. 2016 Oct 6;6:34540. doi: 10.1038/srep34540 (PMC5052591; doi:10.1038/srep34540)
Supplement: Supplementary Information [file srep34540-s1.pdf]

Supplementary Information for:

Beatquency domain and machine learning improve prediction of cardiovascular death after acute coronary syndrome

Yun Liu, Benjamin M. Scirica, Collin M. Stultz, John V. Guttag

Supplementary Methods

Additional Datasets and Outcomes

A total of three datasets were used in this work. The main dataset (D1) that was used to compare frequency and beatquency LF/HF and to train and test machine learning models is described in the body of the manuscript. We employed two additional holdout datasets for further validation of the machine learning models that were developed using dataset D1. The first holdout dataset (D2) consists of 2,255 patients from the treatment arm of the first trial, which contains 77 cardiovascular deaths within one year. This dataset was not merged with D1 because these patients were prescribed ranolazine, a drug that may have anti-arrhythmic properties [3] and thus affect ECG measures. The three datasets, and their uses, are outlined in Supplemental Table S1.

To check that the observations in patients prescribed ranolazine generalize to a broader class of patients, we also evaluated the machine learning models on a second holdout dataset (D3). D3 consists of 765 patients from a different trial, containing 14 cardiovascular deaths within 90 days. We did not split D3 into separate placebo and treatment datasets because, unlike dataset D2, patients in this trial were not prescribed an experimental drug that affects the ECG[2]. This dataset was not merged with D1 because of the shorter duration of follow-up.

Comparing frequency and beatquency bands

To further elucidate the differences between frequency and frequency, we searched for the corresponding frequency bands to our learned beatquency bands in ECG segments with the same heart rate (Supplementary Table S5). For each heart rate, we looked for all five-minute ECG segments with an average heart rate within 1 beats per min, and computed both the frequency and beatquency spectra. We then computed the energy in the two beatquency bands learned from machine learning. Next, we exhaustively searched for the frequency band that is most highly correlated with the two beatquency bands and report the correlation and explained variance.

Supplementary Tables

Supplementary Table S1: Datasets and usages. Basic characteristics of each dataset are presented in Supplementary Table S2.

Dataset	N<br>(# of cardiovascular deaths),<br>median follow-up duration	Usage
D1[1]	2,302 (93), 1 year	(1) Evaluation of HRV-LF/HF measures;<br>(2) Learning and testing of machine learning models (WHRV)
D2[1]	2,255 (77), 1 year	Validation of WHRV
D3[2]	765 (14), 90 days	Validation of WHRV

Supplementary Table S2: Area Under Curve (AUC) averaged over 1,000 test sets taken from dataset D1. Results are reported for all patients ("all") and patients with TRS≤4 ("low TRS") and stratified by gender. Standard error is reported in parenthesis. Bold indicates the highest AUC in each row. Corresponding receiver operating characteristic curves for WHRV and LFHF can be found in Supplementary Fig. S6.

	WHRV		LF/HF		
	Beat	Time	Beat	Time	SDNN
All	**0.7526 (0.0012)**	0.7036 (0.0012)	0.7303 (0.0011)	0.7042 (0.0012)	0.5492 (0.0015)
Low TRS	**0.7730 (0.0015)**	0.7194 (0.0017)	0.7383 (0.0016)	0.6991 (0.0018)	0.5874 (0.0020)
Low TRS, Male	**0.7721 (0.0022)**	0.7344 (0.0024)	0.7279 (0.0023)	0.6925 (0.0025)	0.5569 (0.0028)
Low TRS, Female	**0.7580 (0.0024)**	0.6806 (0.0026)	0.7375 (0.0026)	0.6897 (0.0030)	0.6258 (0.0032)

Supplementary Table S3: Hazard ratio (HR) for cardiovascular death within 1 year in dataset D1 using LF/HF in frequency and beatquency.

	Hazard Ratio (95% CI), p-value	
	Beat	Time
Unadjusted	**4.58 (3.00,6.98), <0.001**	3.65 (2.41,5.53), <0.001
Adj. for TRS	**3.86 (2.51,5.92), <0.001**	3.00 (1.96,4.59), <0.001
Adj for TRS,EF,BNP	**2.38 (1.26,4.51), 0.007**	2.37 (1.26,4.45), 0.006

Supplementary Figures

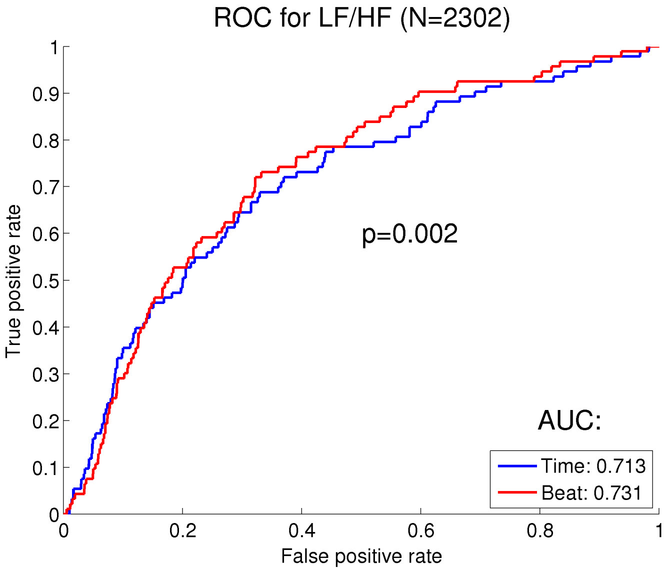

Supplementary Figure S1: Receiver Operating Characteristic (ROC) Curve of LF/HF in frequency and beatquency. The area under the ROC (AUC) is reported in the legend. The difference between the two AUC's is statistically significant (p=0.002). The Kaplan Meier curves are shown in Supplementary Fig. S2.

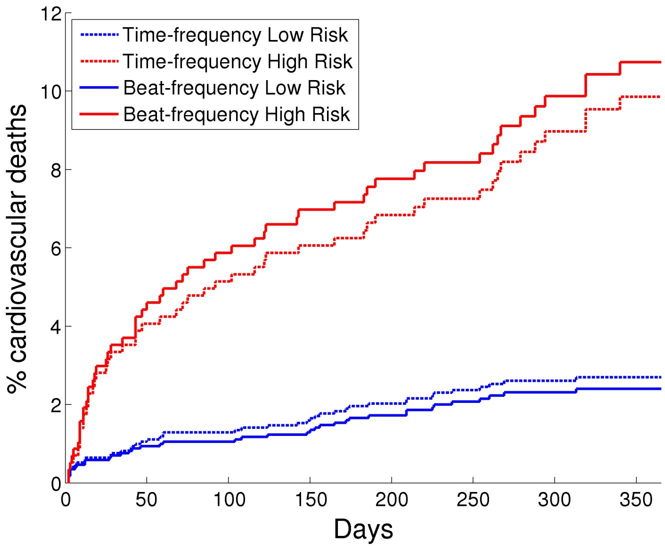

Supplementary Figure S2: Kaplan-Meier curves demonstrating risk stratification using LF/HF in frequency and beatquency. Corresponding hazard ratios are shown in Supplementary Table S3.

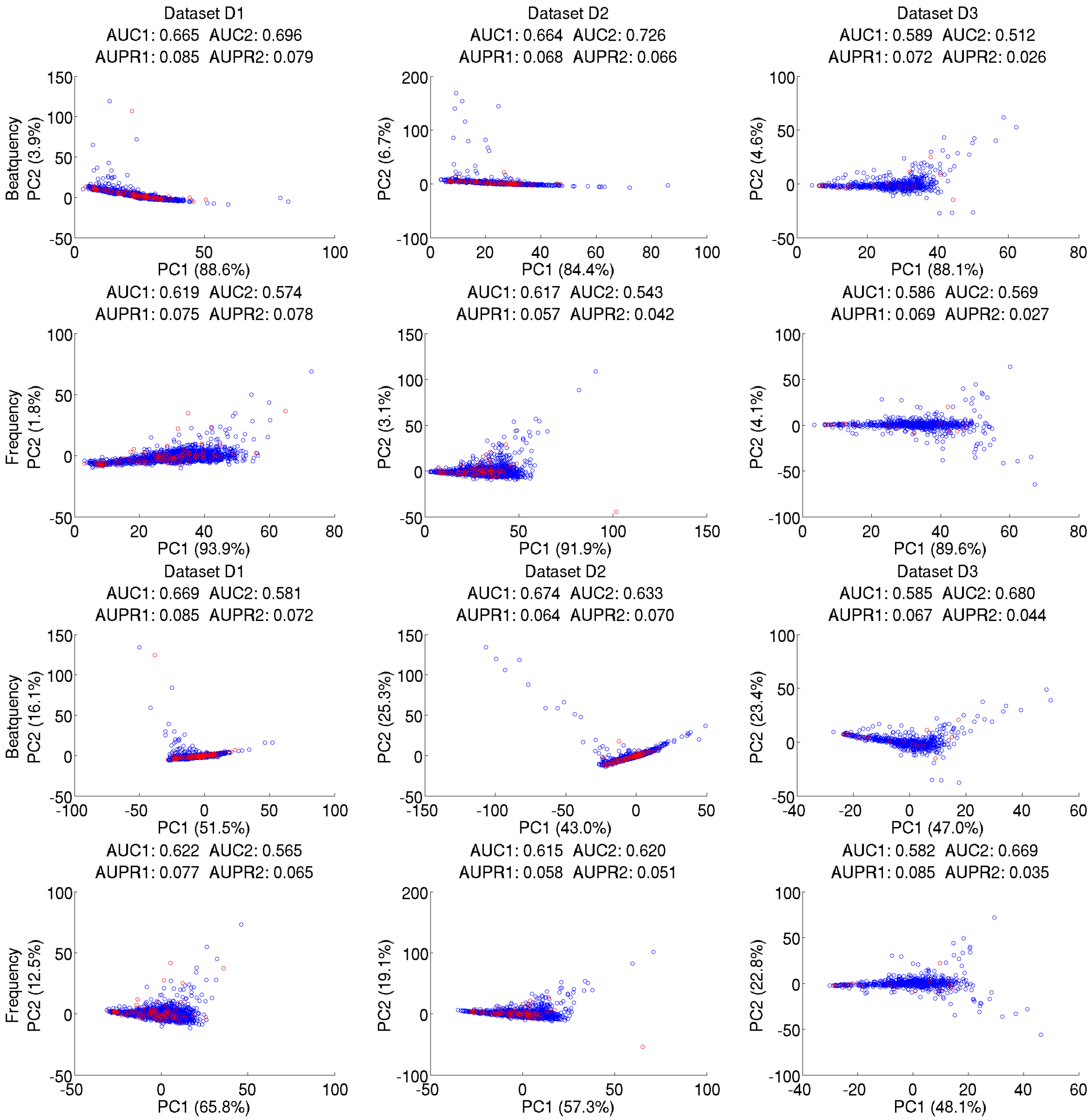

Supplementary Figure S3: Visualizing beat- and frequency features using non-centered (top two rows) and centered (bottom two rows) principle component analysis. Patients who died within one year are in red; patients who did not are in blue.

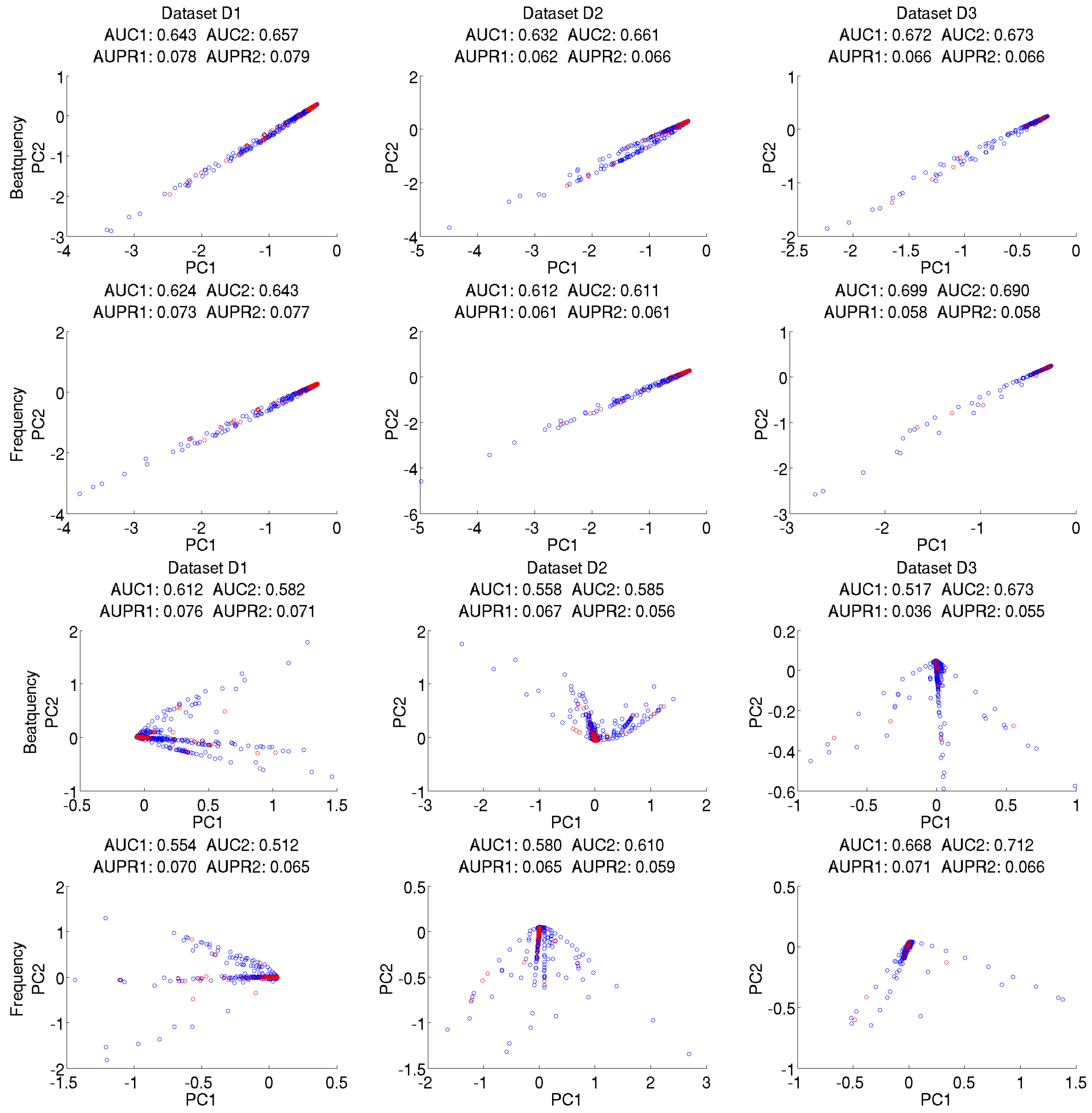

Supplementary Figure S4: Visualizing beat- and frequency features using non-centered (top two rows) and centered (bottom two rows) Minimum Curvilinear Embedding and correlation-based distances. Patients who died within one year are in red; patients who did not are in blue.

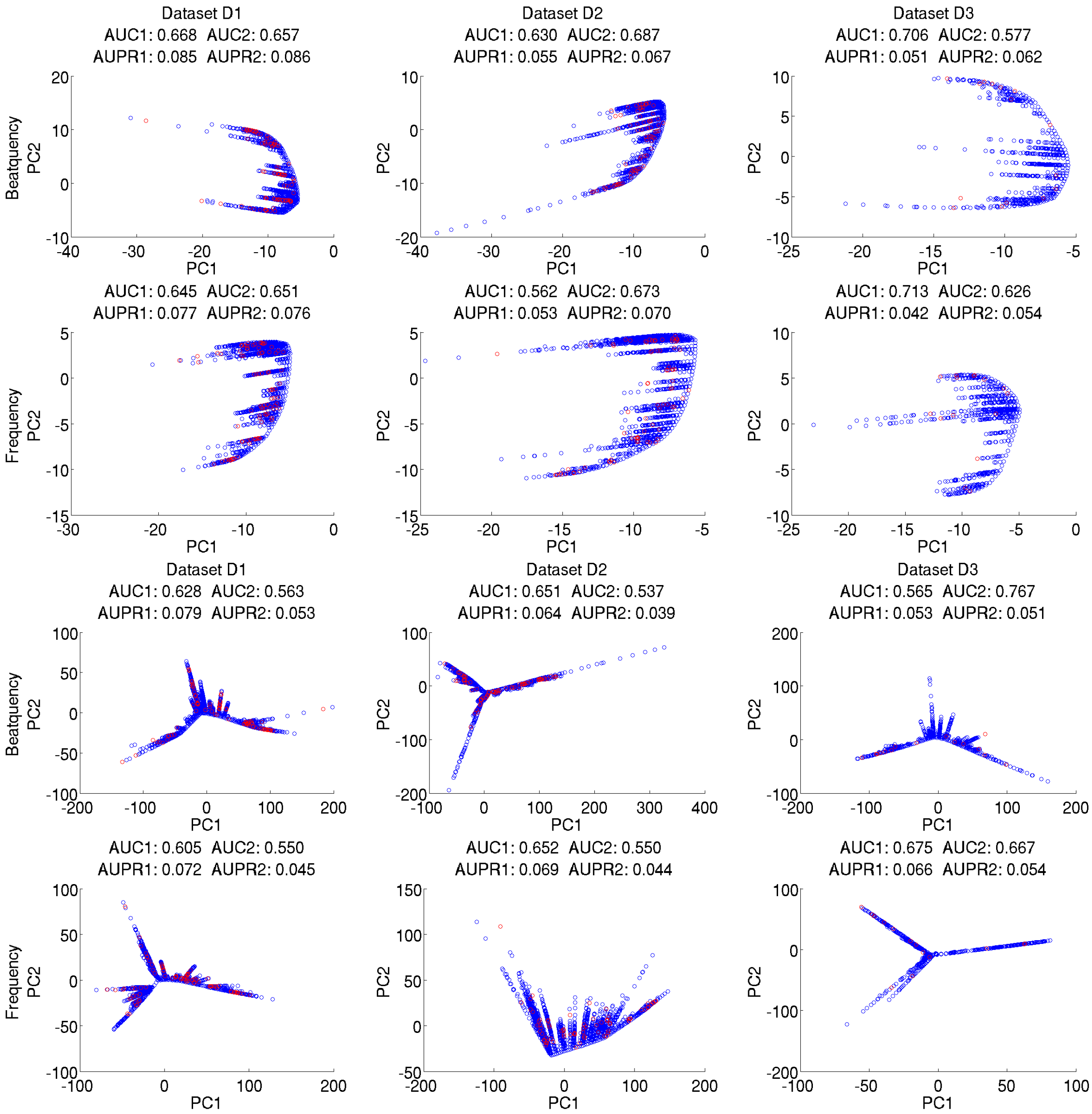

Supplementary Figure S5: Visualizing beat- and frequency features using non-centered (top two rows) and centered (bottom two rows) Minimum Curvilinear Embedding and Euclidean-based distances. Patients who died within one year are in red; patients who did not are in blue.

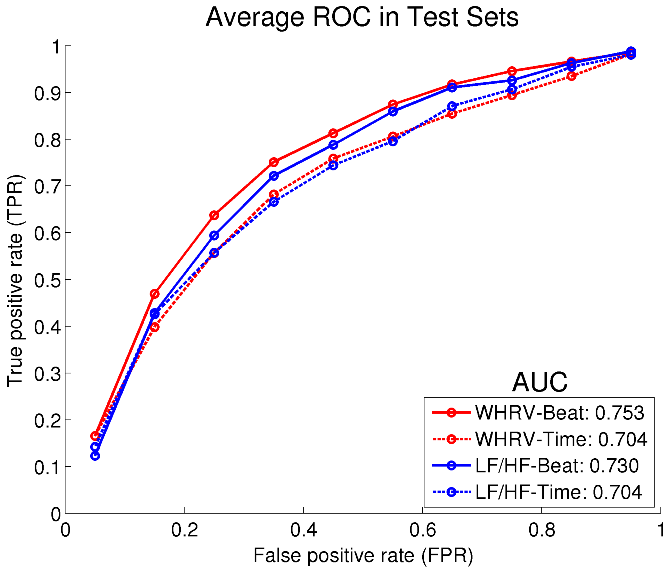

Supplementary Figure S6: Receiver Operating Characteristic (ROC) Curve of WHRV and LF/HF in frequency and beatquency averaged across test sets. The average true positive rate (TPR) values are obtained by first dividing the metric (WHRV or LF/HF) into 10 bins for each test set based on the false positive rate (FPR). Next, the average TPR for each FPR bin is computed across all 1,000 test sets.

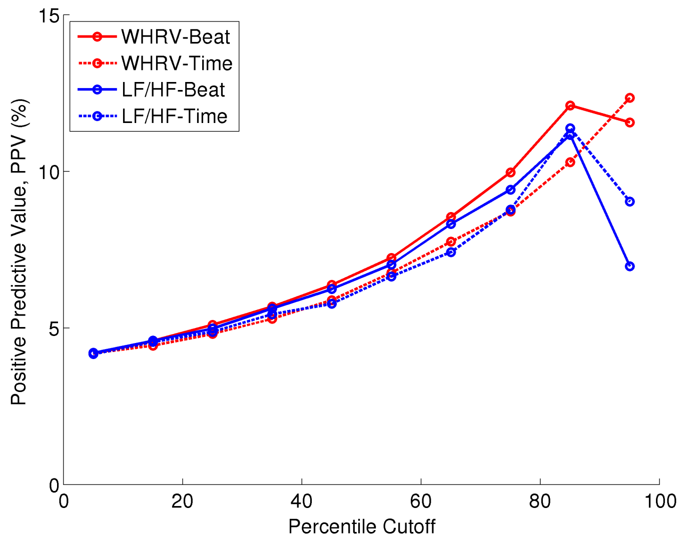

Supplementary Figure S7: Positive predictive values (PPV) for cardiovascular death of WHRV and LF/HF in frequency and beatquency averaged across test sets, using different percentile cutoffs. For comparison, the TIMI Risk Score has PPVs of 5.19% and 9.55% when using the medium and high risk group as cutoffs.

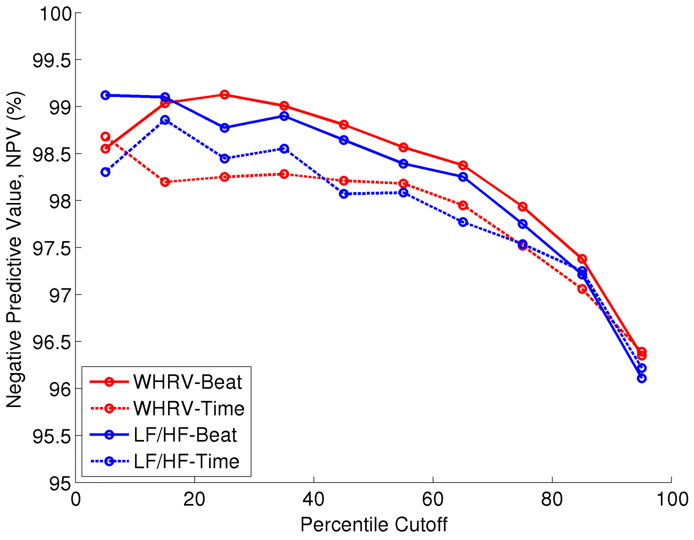

Supplementary Figure S8: Negative predictive values (NPV) for cardiovascular death of WHRV and LF/HF in frequency and beatquency averaged across test sets, using different percentile cutoffs. For comparison, the TIMI Risk Score has NPVs of 99.03% and 97.34% when using the medium and high risk group as cutoffs.

References

1 Morrow, D. A. *et al.* Effects of ranolazine on recurrent cardiovascular events in patients with non–st-elevation acute coronary syndromes. *JAMA: the journal of the American Medical Association* **297**, 1775-1783 (2007).

2 Cannon, C. P. *et al.* Safety, tolerability, and initial efficacy of AZD6140, the first reversible oral adenosine diphosphate receptor antagonist, compared with clopidogrel, in patients with non-ST-segment elevation acute coronary syndrome: primary results of the DISPERSE-2 trial. *Journal of the American College of Cardiology* **50**, 1844-1851, doi:10.1016/j.jacc.2007.07.053 (2007).

3 Scirica, B. M. *et al.* Effect of Ranolazine, an Antianginal Agent With Novel Electrophysiological Properties, on the Incidence of Arrhythmias in Patients With Non–ST-Segment–Elevation Acute Coronary Syndrome Results From the Metabolic Efficiency With Ranolazine for Less Ischemia in Non–ST-Elevation Acute Coronary Syndrome–Thrombolysis in Myocardial Infarction 36 (MERLIN-TIMI 36) Randomized Controlled Trial. *Circulation* **116**, 1647-1652 (2007).

4 Syed, Z. *et al.* Relation of death within 90 days of non-ST-elevation acute coronary syndromes to variability in electrocardiographic morphology. *The American journal of cardiology* **103**, 307-311, doi:10.1016/j.amjcard.2008.09.099 (2009).

5 Liu, Y. *et al.* ECG Morphological Variability in Beat Space for Risk Stratification After Acute Coronary Syndrome. *Journal of the American Heart Association* **3**, e000981 (2014).

6 Fan, R.-E., Chang, K.-W., Hsieh, C.-J., Wang, X.-R. & Lin, C.-J. LIBLINEAR: A library for large linear classification. *The Journal of Machine Learning Research* **9**, 1871-1874 (2008).
